# *Psidium guajava* L*.:* From byproduct and use in traditional Mexican medicine to antimicrobial agent

**DOI:** 10.3389/fnut.2023.1108306

**Published:** 2023-01-24

**Authors:** Daniela Gutierrez-Montiel, Alma L. Guerrero-Barrera, Norma A. Chávez-Vela, Francisco J. Avelar-Gonzalez, Ingrid G. Ornelas-García

**Affiliations:** ^1^Laboratorio de Biología Celular y Tisular, Departamento de Morfología, Centro de Ciencias Básicas, Universidad Autónoma de Aguascalientes, Aguascalientes, Mexico; ^2^Laboratorio de Biotecnología, Departamento Ingeniería Bioquímica, Centro de Ciencias Básicas, Universidad Autónoma de Aguascalientes, Aguascalientes, Mexico; ^3^Laboratorio de Estudios Ambientales, Departamento de Fisiología y Farmacología, Centro de Ciencias Básicas, Universidad Autónoma de Aguascalientes, Aguascalientes, Mexico

**Keywords:** guava, extracts, antimicrobial, phenolic compounds, biofilm, byproducts recovery

## Abstract

Mexico is one of the largest guava producers in the world, so it has access to a huge amount of waste and byproducts obtained after the industrial processing of the fruit. This review discusses the potential recovery of this residue for its application as an antimicrobial agent, considering the phytochemical composition, the bioactivity reported *in-vivo* and *in-vitro*, and the toxicology of the plant. Nowadays there is a growing demand for more natural and safer products, so the use of guava extracts is an interesting initiative, especially due to its availability in the country, its wide variety of traditional uses, and its phytochemical profile. This review highlights the importance and potential antimicrobial use of this plant in today's world.

## 1. Introduction

*Psidium guajava* L. is a native American shrub that can grow in tropical environments around the world (1). It can reach up to 7 m in height and 25 cm in diameter in the trunk when it reaches maturity. Its bark is smooth, thin and coppery brown, while its leaves are green, perennial, coriaceous, and with short petioles. This plant also has white flowers with 4–5 petals and numerous stamens, characteristic of the *Myrtaceae* family (2).

**Figure 1:**
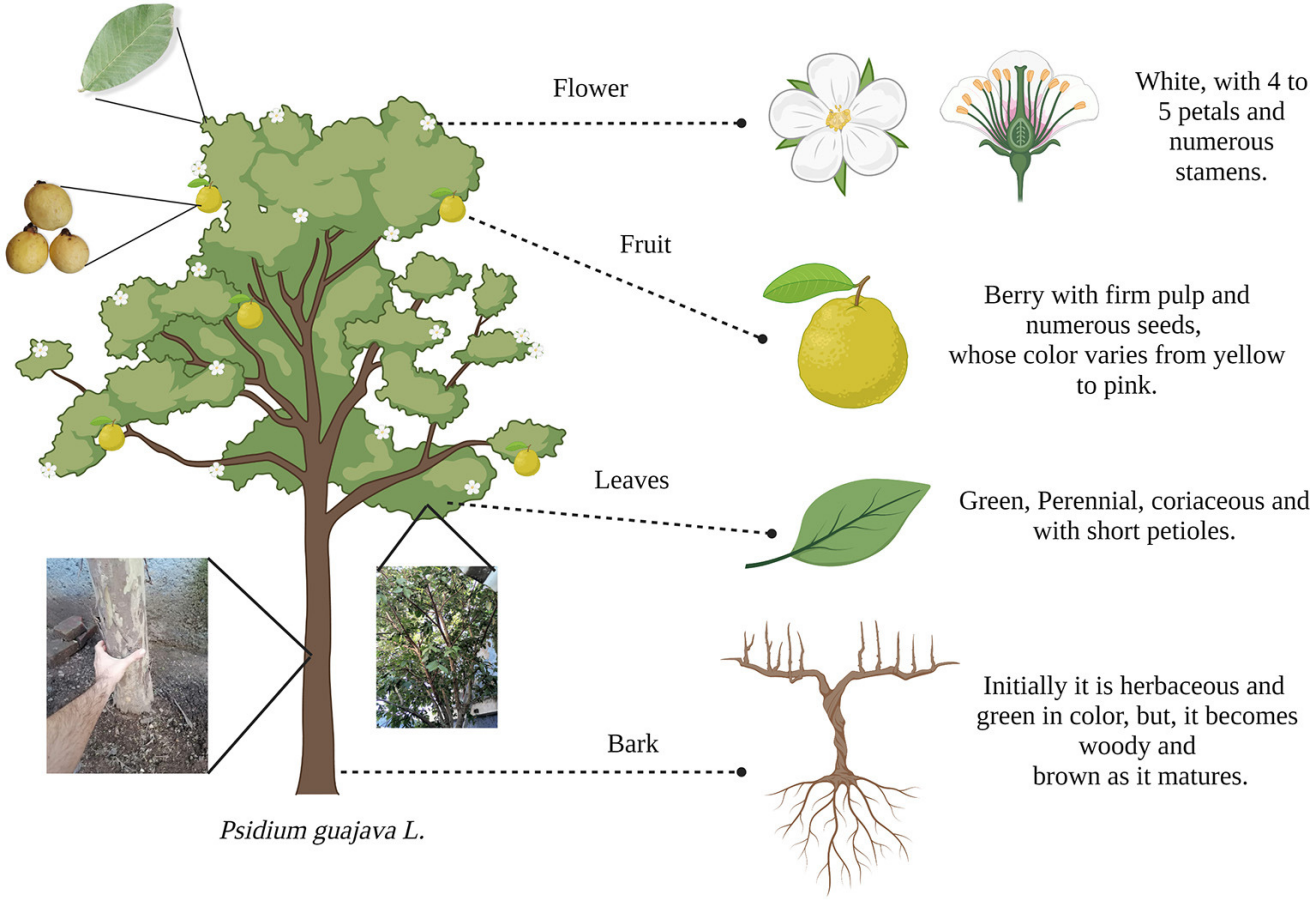
Morphological characteristics of *Psidium guajava* L.

However, its economic importance lies mainly in its fruit, the guava, a juicy berry with numerous seeds and a slightly acid taste. Its shape and color depend on the variety, so we can find guavas from yellow to bright pink and round, ovoid, or pear-shaped (2). The morphological characteristics of *P. guajava* L. are illustrated in Figure 1.

The guava belongs in the *Myrtaceae* family, known for having numerous species with high antioxidant activity (3). On the other hand, the *Psidium* genus has ~ 150 species of shrubs, of which *Psidium guajava* L. is the best known and distributed worldwide (4). Regarding its phenology, this shrub flowers mainly in spring and fruiting can appear throughout the year, mostly during the summer months (5).

Guava worldwide production is estimated to be around 40 million tons. Even if fresh guava trade is limited internationally, the marketing of a wide variety of products derived from its fruit such as preserves, jams, jellies and syrups is becoming more common (6). The highest production of guava can be found in India, Thailand, Brazil, and Mexico (7).

Mexico is a key guava producer in the international market, whose export volume increased more than 150% in < 10 years, from 4,306 tons of guava exported in 2009, to 10,850 tons exported in 2018 (5). Unfortunately, the Mexican agricultural sector presents serious post-harvest problems, generating enormous fruit losses during the different stages of the production chain. Guava is one of the most affected fruits with a waste of more than 50% of its national production (8).

Specifically, in the industrial processing of guava, a heterogeneous mixture of peels, seeds and pulp is generated, and it that can represent up to 30% of the total mass. Guava leaves and bark are other byproducts that are obtained, mainly during the harvest of the fruit (9). Approximately 80 kg of waste per metric ton of fresh fruit is produced during guava processing (10). Unfortunately, and in most cases, the waste generated is thrown into landfills or is incinerated, increasing the environmental load and the total cost of production due to the handling and transportation that these residues require (9, 11).

The aim of this updated review is to expose the potential of *Psidium guajava* L. as an antimicrobial agent to promote the valorization of guava agro-industrial waste and byproducts in Mexico.

## 2. Review methodology

The search engines Google scholar, Elsevier, Springer, MDPI and Science Direct were used to find research articles and reviews related to *Psidium guajava* L. Documents from 2000 to 2022 in Spanish and English were included, focusing in the most recent articles. Articles published before the 2000s were excluded. Book chapters, official sites of the Mexican government and Mexican companies were also consulted. The keywords used were “*Psidium guajava* L.,” “traditional uses,” “phytochemistry,” “biological activities,” “toxicology,” “*In-vivo* evaluation,” and “antimicrobial effects” “diabetes.”

## 3. Traditional uses

**Figure 2:**
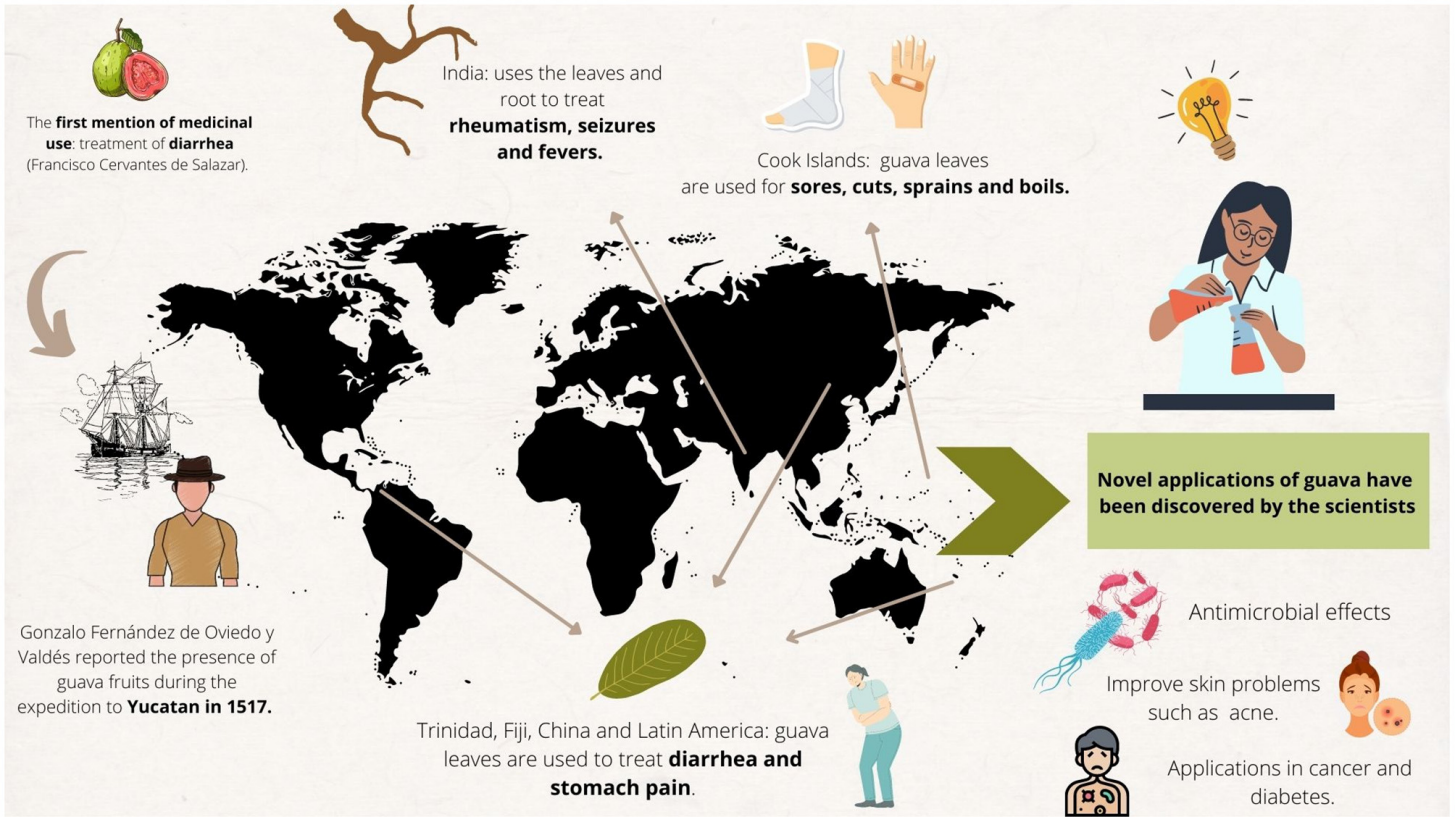
Traditional uses of guava around the world and new perspectives and applications.

*Psidium guajava* L. has been traditionally used as a medicinal plant in different places around the world. Its applications are highly diverse and range from the treatment of gastrointestinal diseases such as vomiting and simple diarrhea to the treatment of wounds, caries, and cough (12–15) as we can see in Figure 2.

All parts of the bush have been used to treat different disorders (16). For example, the leaves and fruit are used to treat respiratory and digestive problems, while the seeds and the leaves have been used as an antispasmodic, anti-inflammatory and even in the control of hypertension and diabetes (17).

Its use also varies depending on geographical location. It has been reported that in Trinidad, Fiji, China and distinct parts of Latin America, guava leaves are used to treat diarrhea and stomach pain (12). On the other hand, in Uruguay they are used to wash the vagina and uterus, especially leucorrhoea cases; in the Cook Islands, guava leaves are used for sores, cuts, sprains, and boils; while in India they are used together with the root to treat rheumatism, seizures, and fevers (12). In the Amazonia this plant is used for the treatment of menstrual disorders, stomach pain, and vertigo, in Cuba, for colds, dysentery, dyspepsia, diarrhea, and hypertension. Finally, in Haiti, guava is used for epilepsy, diarrhea and different skin disorders (15).

Thanks to scientific research, novel applications of guava have been discovered in immune and endocrine systems diseases, as well as in cancer and diabetes. In addition, studies have reported that guava leaf extracts can be used to improve skin problems such as acne and hyperpigmentation (18).

### 3.1. Traditional uses of *Psidium guajava* L. in Mexico

In Mexico, herbalism is an ancient practice that has been used to this day and has great cultural and economic importance. However, in most cases, the active ingredients that provide the beneficial effect are unknown and, therefore, it is a field of study of high scientific interest (19). Guava leaves have been used for medicinal purposes in the country since very remote times, their presence in historical documents on indigenous herbalism has been constant for at least five hundred years (20).

This shrub used to be called by the ancient Mexicans as “*xalxócotl*,” a word in Nahuatl that refers to a fruit that has a “hard and acid shell (*xócotl*) and a sandy texture (*xalli*)”, due to its abundant seeds (20). Guava can be called in diverse ways depending on the Mexican state, for example, in Chiapas, this plant is usually called pata, pocscuy, potok, pox, or sumbadam; in Michoacán it is known as enendi, in Nayarit as caaru, in Morelos as coloc or jaljocote pichi and in Veracruz as asiwit, cuympatan, or pitchcuy (21).

The uses of *P. guajava* L. in the country are as diverse as its names, however, its most common medicinal use is to treat stomach pain. Diarrhea, dysentery, fever, and cough are treated with infusions of guava and its leaves; in the case of skin problems, the leaves are cooked and applied locally. The infusion of guava buds and leaves are used as a de-wormer and guava tea is used to cure scares, using it to give short baths (21).

**Figure 3:**
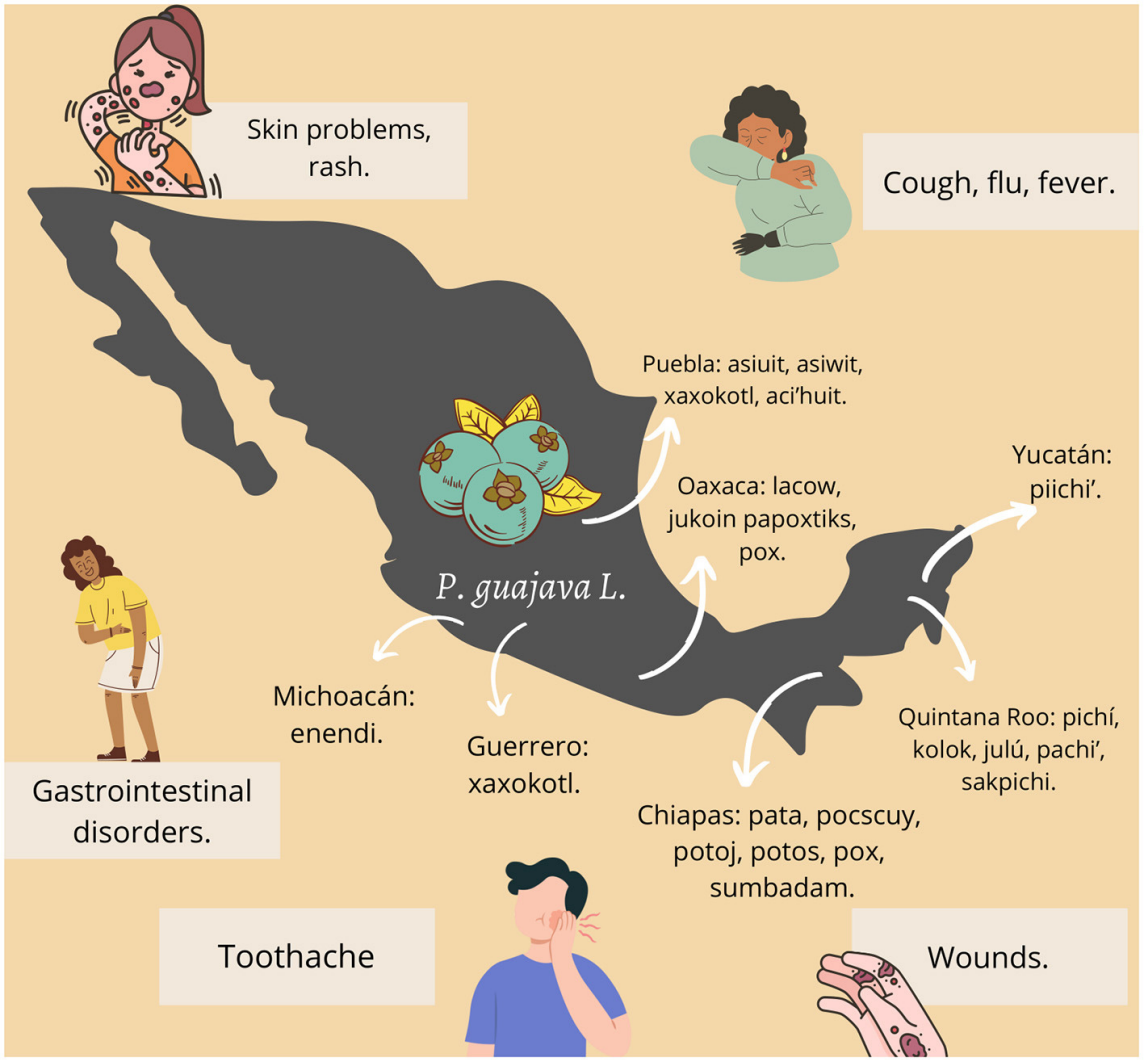
Traditional uses and names of guava in different states of Mexico.

The medicinal use of guava can also vary slightly according to the geographical location in the country, for example, while in southern Veracruz it has been reported that the plant is used to treat diarrhea (22), in the Huasteca Potosina it is also used to treat herpes, wounds, toothache, gastritis and rashes (13). On the other hand, in Guerrero, the infusion of guava leaves is used to cure cough, fever, flu and stomach pain (14). Figure 3 represents and summarizes the different names and traditional uses of guava in Mexico.

## 4. Current importance of guava in Mexico

In Mexico there are twenty entities that harvest this fruit, with Michoacán being the main producing state, followed by Aguascalientes and Zacatecas (23). In 2020, a planted area of 29,872.67 hectares was reported, from which a production of 287,273.02 tons of guava was obtained. This meant an economic spill of $1,664,607.62 billion Mexican pesos (24). The most harvested variety of guava in the country is undoubtedly the “media-china,” while the “china” and the “criolla” are produced in far less quantity (24).

It should be noted that, as of 2008, fresh guava started being sent to the United States of America (25). This is interesting given that, traditionally, the export of this fruit was conducted in its dehydrated or processed versions; however, its nutritional and exotic appeal increased the interest of developed countries like United States, Canada, and Japan (26).

A wide variety of guava processed products such as candies, rolls, ates, ice cream, jams, jellies, juices, and even “guava mole,” a highly seasoned sauce, can be found in Mexico. However, its importance lies not only in its alimentary use, but it also has great relevance for its cosmetic and medicinal applications. In the Mexican market we can also find multiple products that contain guava leaves, like extracts, pills, concentrates, and food supplements that are presented as a natural remedy for stomach pain or as an antioxidant and anti-aging agent. The most relevant example is a drug to relieve the symptoms of colitis called *QG5*, based on a dry extract of *Psidium guajava* L. which is sold in pharmacies throughout the country. The effect of *QG5* is attribute to the presence of quercetin (27).

The composition of the extracts marketed in the country is not clear and, in general, their beneficial effects are attributed to what is already known for its traditional uses. Hence the importance of knowing which compounds are responsible for such effects, and thus be able to move from tradition to science. This will allow us to give a better use to the extract, obtaining a better, and safer beneficial effect and we can even get to know interesting new bioactivities.

## 5. Phytochemical composition

*Psidium guajava* L. chemical composition includes compounds such as tannins, phenols, flavonoids, saponins, carbohydrates, alkaloids, sterols, and terpenoids (28). It is important to consider that the type and abundance of phytochemicals can vary depending on the microclimate and soil conditions of the habitat (29), but also depending on the plant tissue and seasonal changes (30).

The most analyzed part of this shrub is undoubtedly its leaves, given its frequent use as a medicinal remedy. Shabbir et al. (31) reported the approximate composition of guava leaves: 82.47% moisture, 3.64% ash, 0.62% fat, 18.53% protein, 12.74% carbohydrates, 103 mg of ascorbic acid (vitamin C), and 1,717 mg of total phenolic compounds [mg of gallic acid equivalents (GAE)/g]. It should be noted that Shabbir et al. (31) also observed a higher concentration of phenolic compounds in the leaves than in the seeds and fruits of guava.

Guava leaves are also a rich source of vitamins and minerals, such as calcium, potassium, sodium, magnesium, iron, sulfur, vitamin B, and C (32). Even, Thomas et al. (33) mentioned that the leaves have a higher concentration of vitamin B (14.80 mg/100 g), calcium (1,660 mg/100 g), magnesium (440 mg/100 g), phosphorus (360 mg/100 g), and iron (13.50 mg/100 g) compared to the fruits, however, the fruit is richer in vitamin C (228.3 mg/100 g) and potassium (417 mg/100 g).

The higher concentration of calcium in guava leaves (1,660 mg/100 g) compared to the concentration in the fruit (18 mg/100 g) may be since its transpiration rate is higher (34). Similarly, there is a trend toward major magnesium allocation in transpiring organs such as leaves (440 mg/100 g) and flowers rather than roots and fruits (22 mg/100 g) (35).

Furthermore, it is important to consider that the nutrient content varies considerably between different plant organs, as well as by the age of the tissue in question. Beyond that, the nutrient content largely depends on optimal uptake and soil quality (36).

The polysaccharides found in guava leaves have also had great relevance in recent years since they have been found to be beneficial for the treatment of diabetes mellitus symptoms, as will be discussed in more depth later, and they can also be used as antioxidant additives in food (37).

Díaz-de-Cerio et al. (38) identified more than 70 phenolic compounds in guava leaves by HPLC-DAD-QTOF-MS using hydro-alcoholic extracts obtained by sonication. Table 1 shows the main compounds identified in extracts of *Psidium guajava* L. reported by different authors.

**Table 1:** Phytochemical composition of different extracts of *Psidium guajava* L.

**Method**	**Extract**	**Location**	**Quantity of detected compounds**	**Main identified compounds**	**References**
UPLC-ESI-QTOF-MS	Tannic fraction of dried leaves	South of Ceará, Brazil	10	Vescalagin, Catechin, Quercetin, Guavinoside C, 2,6-dihydroxy-3-methyl-4- O-(6"-O-galloyl-β-Dglucopyranosyl)- benzophenone (1S*,5S*)-2,2-Bis(biphenyl-4-yl)-5-indian-1- yltetrahydrofuran(IV-81)	(39)
UPLC-ESI-QTOF-MS	Flavonoid fraction of dried leaves	South of Ceará, Brazil	11	Catechin, Ellagic acid, Reynoutrin, Guajaverin, Myrciaphenone B, Guavinoside B, Morin	(39)
GC-MS	Leaves essential oil	Rio Verde in Goiás, Brazil	17	Limonene, 1,8-Cineole, α-Copaene, β-Caryophyllene, α-Humulene, 4,11-Selinadiene, γ-Muurolene, Aromadendrene oxide, δ-Selinene, α-Panasinsene, *trans*-Nerolidol, β-Caryophyllene oxide, Humulene epoxide II, Longipinene epoxide, *epi*-α-Muurolol, Selin-11-en-4α-ol, α-Cadinol	(40)
GC-MS	Leaves essential oil	El-Behera Governorate, Egypt	10	α -Pinene, Benzaldehyde, ρ -Cymene, Limonene, 1, 8-Cineole, β -*cis*-Ocimene, γ -Terpinene, α -Terpineol, β -Caryophyllene, α -Humulene	(41)
GC-MS	Leaves ethyl acetate extract	Vadlamudi, India	25	Caryophyllene, α-Copaene, *cis*-Muurola-3,5-diene, Humulene, Cyclosativene, *cis*-à-Bisabolene, Spathulenol, Cubenol, Torreyol, α-Cadinol, α-Bisabolol, Isoamyllaurate, Bicyclo[5.3.0]decane, 2-methylene-5-(1-methylvinyl)-8-methyl-5,6,6-Trimethyl-5-(3-oxobut-1-enyl)-1-oxaspiro[2.5]octan-4-one	(42)
GC-MS	Guava seed oil	Punjab, India	33	N-Acetylethylenediamine, Cyclobutylsilane, Oxirane, Valeric acid, 2-Heptenal, D-Limonene, Decane, 2,6-Dimethyl-6-nitro-2-hepten-4-one, 2,4-Nonadienal, 4,7-dimethyl Undecane, 9-methyl-1-undecane, Cubebene, 2,4-dihydroxy-6-methyl Benzaldehyde, Caryophyllene, Caryophyllene oxide, 2,4,6-trimethyl octane, 6,9-Heptadecadiene, 2,7,10-trimethyl dodecane, n-Hexadecanoic acid, 2-Chloroethyl linoleate, Octadecanoic acid, 7-Nonenamide	(43)
GC-MS	Methanolic pulp extract (industrialized liquid pulp with food additives like BHT)	Brazil	36	L-5-Propylthiomethylhydantoin, Ethanediamide, L-Alanine, methyl ester, Sulfur tetrafluoride, Pentanoic acid, 3-methyl-4-oxo, Isobutyl acetate, 2-Ethoxytetrahydrofuran, Furfural, 2-Azetidinone, 1-phenyl-, Ethylbenzene, Butyrolactone, 1,2-Propanediol, diacetate, Propanedioic acid, diethyl ester, 1,4-Butanediol, diacetate, Benzoic acid, Benzaldehyde, 2,4-dimethyl, 5-Hydroxymethylfurfural, 1,5-Diacetoxypentane, 5-Acetoxymethyl-2-Furaldehyde, Pentadecane, Hexadecanoic acid, ethyl ester, 7-Octadecenoic acid, methyl ester 7-Tetradecenal	(44)
GC-MS	Fruit essential oil	Pakistan	38	1R- α-Pinene, Decane, D-Limonene, Eucalyptol, Globulol, Caryophyllene, Tau-cadinol, Nerolidol, Copaene α-Cadinol, aromandendrene, Humulene (Z,Z)-2,6-farnesol *cis*-α-Bisabolene, β-Bisabolene *epi*-Globulol α-Bisabolol, α-Selinene	(45)
GC-MS	Leaves essential oil	Pakistan	39	Caryophyllene Nerolidol 2 Aromadendrene *cis*-α-bisabolene Tetracosane Octadecane Z,Z,Z-1,5,9,9-tetramethyl-1,4,7-cycloundecatriene β-bisabolene Limonene Octacosane δ-cadinene, 1,4-cadadiene β-caryophyllene	(45)
GC-MS	Flowers essential oil	Rio Verde in Goiás Brazil	13	*trans*-β-Caryophyllene α-Humulene Nerolidol β-Selinene α-Selinene Germacrene D δ-Selinene Caryophyllene oxide Spathulenol Globulol Cubenol *epi*-α-Cadinol α-Cadinol	(46)

The recovery of the unused flesh, peels, seeds, flowers, bark, and leaves is a great opportunity to guava producing countries, considering the variety of bioactive compounds that can be extracted (47).

Nonetheless, an aspect to consider during the use of industrial byproducts is the possible presence of food additives, as is the case of the pulp extract used by dos Santos et al. (44), which contained different additives, among them, the antioxidant BHT, which may cause allergies, eczema, rash, and angioedema, therefore BHT cannot exceed an ADI of 0.5 mg/kg (48). As the presence of these additives probably remains after the extraction and can affect the activity of the extract, it is important to determine in what concentrations are they found in the plant material and what are the possible toxicological effects that they may cause depending on the route of administration.

On the other hand, several authors have reported that the main component of the extracts or essential oils of guava leaves is β-Caryophyllene (45, 49, 50), which has recently gained attention due to its potential application for the treatment of various disorders such as cancer, chronic pain, and inflammation (51). In addition, many of the plant extracts where β-Caryophyllene is found have antimicrobial effects, notwithstanding the role that this compound plays in said activity is still unclear. One of the proposed modes of action is that it alters the permeability of the bacterial membrane causing the formation of non-selective pores (52).

Even if the mechanism of action and the interactions between all the components of the *Psidium guajava* L. extracts is ambiguous, many researchers around the world have identify different beneficial effects of their components, for example, their anti-inflammatory, antioxidant, and antimicrobial activity. Table 2 shows some properties attributed to compounds found in the different guava extracts.

**Table 2:** Bioactivity of different compounds found in *Psidium guajava* L. extracts.

**Compound**	**Classification**	**Activity**	**References**
Quercetin	Phenolic compound	Antioxidant, anti-inflammatory and anti-allergy	(53)
Gallic acid	Phenolic compound	Antibacterial, anti-fungal, antiviral, anti-inflammatory, antioxidant, anticarcinogenic, anti-diabetic	(53)
β-Caryophyllene	Sesquiterpene	Local anesthetic, anticarcinogenic, antioxidant, antibiotic, anti-inflammatory, neuroprotective anxiolytic, antidepressant, and anti-alcoholism	(45)
Copaene	Sesquiterpene	Anti-inflammatory and *in- vitro* anti-tumor activity	(54)
Limonene	Terpene	Anti-inflammatory and *in-vitro* anti-tumor activity	(54)
Catechin	Phenolic compound	Antioxidant, prevention or reduction of skin damage, activation of collagen synthesis, and inhibition of the production of matrix metalloproteinase enzymes. Anti-microbial, anti-viral, and anti-inflammatory	(55)
Ellagic acid	Phenolic compound	Anti-inflammatory, anti-microbial, antioxidant	(56)
Humulene	Sesquiterperne	Anti-tumor, anti-microbial, and anti-inflammatory. Great gastroprotective, cicatrizing, analgesic, and antioxidant potentials	(57)
Nerolidol	Sesquiterperne	Antioxidant, anti-inflammatory, and anti-microbial. It also enhances skin penetration and permeation	(58)
α-Pinene	Terpene	Anticarcinogenic, anti-inflammatory, and anti-allergy	(59)
Eucalyptol	Terpene	Insecticide, anti-fungal, anti-microbial, anti-inflamatory, and gastroprotective effect	(60)
Guajaverin	Phenolic compound	Anti-viral and anti-bacterial activity	(61, 62)
Bisabolol	Sesquiterpene	Anti-inflammatory, antispasmodic, anti-allergic, and vermifuge properties	(63)
α-Cadinol	Sesquiterpene	Anti-bacterial and anti-fungal	(64)

Observing the properties of some of its components (Table 2), it is not surprising that numerous studies have reported the potential of different extracts of *Psidium guajava* L. as an antimicrobial agent, so in this section we will dedicate ourselves to present the most relevant discoveries of recent times on this topic to create an overview of the different applications that we can get from this plant and what needs to be done to make these benefits available to society.

## 6. Antimicrobial effects of *Psidium guajava* L.

We are currently facing a serious global problem regarding microbial resistance, which has been aggravated during the COVID-19 pandemic. In Mexico, it has already been reported that strains such as *Staphylococcus aureus, Klebsiella pneumoniae* and *Enterococcus faecium* increased their resistance during the pandemic (65). Consequently, there is an urgent need for new and better antimicrobials, where the use of phytochemicals has drawn the attention of many researchers around the world.

Table 3 summarizes various evaluations of the antimicrobial effect of *Psidium guajava* L. carried out in recent years. Different solvents, plant parts and tests were used. Overall, the results indicate that guava extracts have potential activity on multiple bacteria strains and on yeasts of the genus *Candida*.

**Table 3:** Antimicrobial effects of different extracts of *Psidium guajava* L.

**Microorganism**	**Method**	**Anatomical part**	**Extract**	**Results**	**References**
*Candida albicans*	96-well plate broth microdilution method (the concentrations in the wells ranged from 2 to 2,048 μg/mL) Standard: fluconazole	Leaves	Tannic fraction	*IC*_50_(μg/mL) = 69.29 ± 1.89	(39)
			Flavonoid fraction	*IC*_50_(μg/mL) = 2,690.52 ± 3.18	
			Tannic + flavonoid fraction	*IC*_50_(μg/mL) = 207.8 ± 4.28	
*Candida tropicalis*	96-well plate broth microdilution method (the concentrations in the wells ranged from 2 to 2,048 μg/mL) Standard: fluconazole	Leaves	Tannic fraction	*IC*_50_(μg/mL) = 188.9 ± 2.59	(39)
			Flavonoid fraction	*IC*_50_(μg/mL) = 3,444.62 ± 3.47	
			Tannic + flavonoid fraction	*IC*_50_(μg/mL) = 315.1 ± 4.41	
*Candida krusei*	96-well plate broth microdilution method (the concentrations in the wells ranged from 2 to 2,048 μg/mL) Standard: fluconazole	Leaves	Tannic fraction	*IC*_50_(μg/mL) = 261.7 ± 3.25	(39)
			Flavonoid fraction	*IC*_50_(μg/mL) = 1,199.57 ± 3.66	
			Tannic + flavonoid fraction	*IC*_50_(μg/mL) = 115.7 ± 2.58	
*Streptococcus salivarius*	96-well plate broth microdilution method (the concentrations in the wells ranged from 50 to 400 μg/mL) Standard: chlorhexidine digluconate	Fresh leaves	Essential oil	MIC (μg/mL) = 400	(40)
*Streptococcus mutans*	96-well plate broth microdilution method (the concentrations in the wells ranged from 50 to 400 μg/mL) Standard: chlorhexidine digluconate	Fresh leaves	Essential oil	MIC (μg/ml) = 200	(40)
*Streptococcus mitis*	96-well plate broth microdilution method (the concentrations in the wells ranged from 50 to 400 μg/mL) Standard: chlorhexidine digluconate	Fresh leaves	Essential oil	MIC (μg/ml) = 200	(40)
*Streptococcus sanguinis*	96-well plate broth microdilution method (the concentrations in the wells ranged from 50 to 400 μg/mL) Standard: chlorhexidine digluconate	Fresh leaves	Essential oil	MIC (μg/ml) = 400	(40)
*Streptococcus sobrinus*	96-well plate broth microdilution method (the concentrations in the wells ranged from 50 to 400 μg/mL) Standard: chlorhexidine digluconate	Fresh leaves	Essential oil	MIC (μg/ml) = 100	(40)
*Propionibacterium acnés* (now *Cutibacterium acnes)*	96-well plate broth microdilution method (the concentrations in the wells ranged from 20 to 2,500 μg/mL) Standard: tetracycline	Leaves	Essential oil	*IC*_50_(μg/mL) = 309 MIC (μg/mL) = 321	(66)
*Staphylococcus epidermidis*	96-well plate broth microdilution method (the concentrations in the wells ranged from 20 to 2,500 μg/mL) Standard: tetracycline	Leaves	Essential oil	*IC*_50_(μg/mL) = 416 MIC (μg/mL) = 486	(66)
	Broth dilution method (concentrations ranging from 40,000 to 160,000 μg*l*mI) Recovery of medium and streaking in solid medium to determine the MBC Standard: nutrient broth without extract	Leaves	Methanolic extract	MIC (μg/ml) = 20,000 ± 0.3 MBC (μg/ml) = 80,000 ± 0.2	(67)
			Ethyl acetate extract	MIC (μg/ml) = 20,000 0 ±0.0 MBC (μg/ml) = 40,000 ± 0.3	
*Streptococcus gordonii*	Disc diffusion test and 96-well plate broth microdilution method (the concentrations in the wells ranged from 200 to 50,000 μg/mL) Standard: chlorhexidine 0.12%	Leaves	Chloroformic residue of *P. guajava L*. crude extract (50,000 μg/mL)	Mean diameters of inhibition halos (mm) = 9.12 MIC (μg/mL) = 780	(28)
*Staphylococcus aureus*	Agar dilution method (concentrations ranging from 625 to 10,000 μg/mL)	Dried powdered plant	Ethanolic extract	MIC (μg/ml) = 1,250	(68)
	Agar dilution method	Leaves	Essential oil	MIC (μg/ml) = 6.75	(41)
	96-well plate broth microdilution method (the concentrations in the wells ranged from 7.8 to 1,000 μg/mL) Recovery of medium and streaking in solid medium to determine the MBC Standard: extracts were used as negative controls	Pulp (agroindustrial waste)	Methanolic extract	MIC (μg/ml) = 31.25 MBC (μg/ml) = 62.5	(44)
*Escherichia coli*	Agar dilution method (concentrations ranging from 625 to 10,000 μg*l*mI)	Dried powdered plant	Ethanolic extract	MIC (μg/ml) = 625	(68)
	Broth dilution method (concentrations ranging from 40,000 to 160,000 μg*l*mI) Recovery of medium and streaking in solid medium to determine the MBC Standard: nutrient broth without extract	Leaves	Methanolic extract	MIC (μg/ml) = 40,000 ± 0.5 MBC (μg/ml) = 80,000 ± 0.1	(67)
			Ethyl acetate extract	MIC (μg/ml) = 40,000 ± 0.0 MBC (μg/ml) = 80,000 ± 0.1	
*Salmonella Enteritidis*	Agar dilution method (concentrations ranging from 625 to 10,000 μg*l*mI)	Dried powdered plant	Ethanolic extract	MIC (μg/ml) = 625	(68)
*Pasteurella multocida*	Agar dilution method (concentrations ranging from 625 to 10,000 μg*l*mI)	Dried powdered plant	Ethanolic extract	MIC (μg/ml) = 5,000.00	(68)
*Mycoplasma gallisepticum*	Agar dilution method (concentrations ranging from 625 to 10,000 μg*l*mI)	Dried powdered plant	Ethanolic extract	MIC (μg/ml) = 1,250.00	(68)
*Bacillus cereus*	Broth dilution method (concentrations ranging from 40,000 to 160,000 μg*l*mI) Recovery of medium and streaking in solid medium to determine the MBC Standard: nutrient broth without extract	Leaves	Methanolic extract	MIC (μg/ml) = 40,000 ± 0.1 MBC (μg/ml) = 40,000 ± 0.4	(67)
			Ethyl acetate extract	MIC (μg/ml) = 40,000 ± 0.2 MBC (μg/ml) = 80,000 ± 0.3	
*Pseudomonas aeruginosa*	Broth dilution method (concentrations ranging from 40,000 to 160,000 μg*l*mI) Recovery of medium and streaking in solid medium to determine the MBC Standard: nutrient broth without extract	Leaves	Methanolic extract	MIC (μg/ml) = 40,000 ± 0.7 MBC (μg/ml) = 80,000 ± 0.0	(67)
			Ethyl acetate extract	MIC (μg/ml) = 40,000 ± 0.1 MBC (μg/ml) = 80,000 ± 0.1	

It should be noted that a proper comparison between studies cannot be made, even if similar methodologies were used, since each author established the MIC differently, for example, Soliman et al. (41) defined MIC as the lowest concentration that had granular appearing micro-colonies of growth instead of filamentous radiating colonies on solid agar; while for Hemeg et al. (68) MIC was the lowest concentration of extract that resulted in no visible growth on the surface of the agar. In addition, we can also find multiple differences between the methods to measure the antimicrobial effect, for example, dos Santos et al. (44) used a 0.1% resazurin solution, while Festus et al. (67) analyzed bacterial growth based on turbidity.

Due to all these variants, it is difficult to interpret the results in a general way and the fact that different solvents, types of extraction, and parts of the plant were used, makes everything more complex, because the results depend on a great variety of factors. The selection of controls is also a factor to improve, given that in some studies negative controls were used but not positive ones and vice-versa. In addition, the part of the plant used to perform the extraction of phytochemicals and the location where the plant material was collected should be clear and precise.

However, the highest dose used in the studies analyzed was 160 mg/ml (160,000 ug/ml) by Festus et al. (67), but the highest reported MIC was 40 mg /ml (40,000 ug/ml) for the bacteria *Pseudomonas aeruginosa, Bacillus cereus, and Escherichia coli* (67).

### 6.1. Anti-biofilm effects of guava extracts

Biofilms are architectural elements embedded in self-produced extracellular polymeric substances that adhere to inert or biological surfaces. These elements allow microorganisms to adapt to their environment and on certain occasions give them the ability to escape host defense systems and may even confer resistance to antibiotics given the difficult penetration of molecules into the matrix of extracellular polymeric substances (69).

Therefore, finding substances that can inhibit the formation and growth of biofilms is of special interest, especially if they are substances of natural origin, such as phytochemicals, which can be substitutes for synthetic drugs without presenting significant secondary effects (28).

Unfortunately, recent studies of the potential antibiofilm activity of *P. guajava* L. are not numerous and focus on the effect on *S. aureus*. Therefore, research on multiple microorganisms is needed to evaluate the spectrum of antibiofilm activity of guava extracts. Table 4 presents a summary of the results obtained and the possible mechanism of action proposed by the authors.

**Table 4:** Potential antibiofilm activity of different *Psidium guajava* L. extracts.

**Microorganism**	**Extract**	**Results**	**Possible mechanism of action**	**References**
*Pseudomonas aeruginosa PAO1*	Flavonoid fraction of guava leaves	Anti-*quorom sensing* activity in a *C. violaceum* CV026 biosensor bioassay	The flavonoid fraction interferes *quorom sensing* by inhibiting the response to the autoinducer (N-acyl homoserine lactone). Compounds that probably are responsible for this effect are quercetin and quercetin-3-O-arabinoside	(70)
*Staphylococcus aureus* clinical isolates and ATCC 25923	Benzyl isocyanate isolated from the leaves of *Psidium guajava* L.	MBIC (μg/ml) = 440–870 MBEC (μg/ml) = 1,000–2,100	Benzyl isocyanate induces the production of poor-quality extracellular polymeric substances and can inhibit major biofilm regulatory molecules of *S. aureus*	(69)
*Staphylococcus aureus*	Pulp methanolic extract (agroindustrial waste)	MBEC (μg/ml) = 250	The presence of polyphenols and compounds like L-5-Propylthiomethylhydantoin, that has bacteriostatic activity	(44)
*Staphylococcus aureus*	Petroleum ether guava leaves extract	Dose: 1,000 μg/ml Percentage of biofilm inhibition: 25.2 ± 0.53%	Flavonoids prevent the correct transmission of signals, leading to shutdown of *quorum sensing*. In addition, terpenoids alter the fatty acid composition of the cell membrane, which causes the hydrophobicity of cells leads to biofilm eradication	(71)
		Dose: 2,000 μg/ml Percentage of biofilm inhibition: 62.9 ± 0.48%	
	Ethanolic guava leaves extract	Dose: 1,000 μg/ml Percentage of biofilm inhibition: 76.83 ± 0.56%		
		Dose: 2,000 μg/ml Percentage of biofilm inhibition: 80.0 ± 0.86%		

The MBEC (Minimum Biofilm Eradication Concentration) reported by dos Santos et al. (44) (250 μg/ml) is lower than those reported by Dutta et al. (69) (1,000–2,100 μg/ml). This could indicate that probably the guava leaf extract has a higher activity compared to the compound Benzyl isocyanate (isolated from guava leaves) on its own. The interaction of the components of the extract and a possible synergy may be involved in this fact, but future research is needed to clarify and verify this issue.

The antibiofilm mechanism exhibit by the phytochemicals presents in plant extracts are highly diverse, and among them are the decrease in the production of virulence factors, the inhibition of the formation of the polymeric matrix, the suppression of cell adhesion and the alteration of *quorum sensing* (72).

The lack of antibiofilm evaluations with extracts from another part of the plant, since those published focus mainly on the leaves and their derivatives, shows that there is still much to discover on this topic and a large field of study opens for those interested in this area.

### 6.2. Antiviral effects of *Psidium guajava* L.

Nowadays, researchers have been particularly interested in the study of medicinal plants as a therapeutic option for the treatment of viral infections, based on traditional ethnomedical wisdom and taking advantage of their molecules to develop antiviral drugs (73). Different studies have revealed that *Psidium guajava* L. extracts may have antiviral activity and some of them are summarized below.

The infusion of guava leaves was able to inhibit the growth of isolated influenza A (H1N1) virus. Likewise, it was able to inhibit viral hemagglutination and sialidase activity (*IC*_50_= 0.44 ± 0.05–7.50 ± 0.49) (74). Therefore, it appears to be effective for the control of pandemic and epidemic influenza viruses, including oseltamivir-resistant strains (75).

Sharma et al. (76) reported that the guava leaves extract and its nanoparticles reduce/stop chikungunya virus replication in the Vero cell line. Specifically, the authors reported a viability of 84.21% of the cells treated with the guava extract, a viability of 64.40% of the cells treated with the nanoparticles of the extract, and a viability of 24.03% of the positive control (virus + cells). The authors conclude that in the absence of vaccines and antivirals, the extracts of *P. guajava L*. can be an alternative treatment.

The antiviral effect of the crude aqueous extract of 10 plants used in traditional medicine in the Philippines, including the leaves of *Psidium guajava* L., was analyzed by Vista et al. (77). The authors reported that guava leaves were one of the best candidates against ZIKA virus (ZIKV) along with *M. charantia, V. negundo*, and *B. balsamifera*.

Trujillo-Correa et al. (78) reported that four compounds isolated from guava bark extracts, specifically gallic acid, quercetin, naringin, and catechin, inhibited dengue virus (DENV-2) replication. Of these, catechin was the most promising compound. The authors also mention that the anti-DENV effect of the *P. guajava* L. extract could be related to the inhibition of the enzyme α-glucosidase, since it has been described as essential for the correct folding of viral glycoproteins and for the assembly of the virion.

Shin et al. (79) studied the potential of BEN815, a natural nutraceutical composed of extracts of guava leaves (*Psidium guajava* L.), green tea leaves (*Camellia sinensis*), and rose petals (*Rosa hybrida*) to treat COVID-19. The authors reported that BEN815 showed antiviral activity against SARS-CoV-2, with an *IC*_50_= 34.38 μg/ml. In addition, it is mentioned that of the compounds found in the nutraceutical, EGCG (Epigalocatechin gallate) plays a key role in the antiviral effect against SARS-CoV-2.

## 7. Potential mechanisms of action of antimicrobial activity

The exact mechanism of antibacterial activity is still not clear, mainly because phytochemicals have highly variable structures, generating multiple possible modes of action. Moreover, plant extracts contain a complex mixture of compounds whose interaction can influence the mechanism (80). Therefore, the mode of action depends on the type of extract or essential oil and the microorganism used (81).

Nonetheless, it has been shown that phenolic compounds can interact with bacterial cell walls, leading to their rupture and the release of cellular components (80), they can also suppress several microbial virulence factors (among them biofilm formation and toxin production), inhibit the synthesis of nucleic acids and the activity of enzymes (82). In addition, it has been reported that gram-negative bacteria are more resistant to phenolic compounds, probably due to the presence of an outer membrane and enzymes in the periplasmic space that can damage molecules that enter the bacteria (80).

Similarly, the mode of action of terpenes, also an important component of guava extracts, remains largely unknown, however, it has been observed that most terpenoids can inhibit two essential processes for microbial survival: oxygen uptake and oxidative phosphorylation. Thus, terpene interaction leads to alteration in cellular respiration which later causes uncoupling of oxidative phosphorylation in the microbe (83).

**Figure 4:**
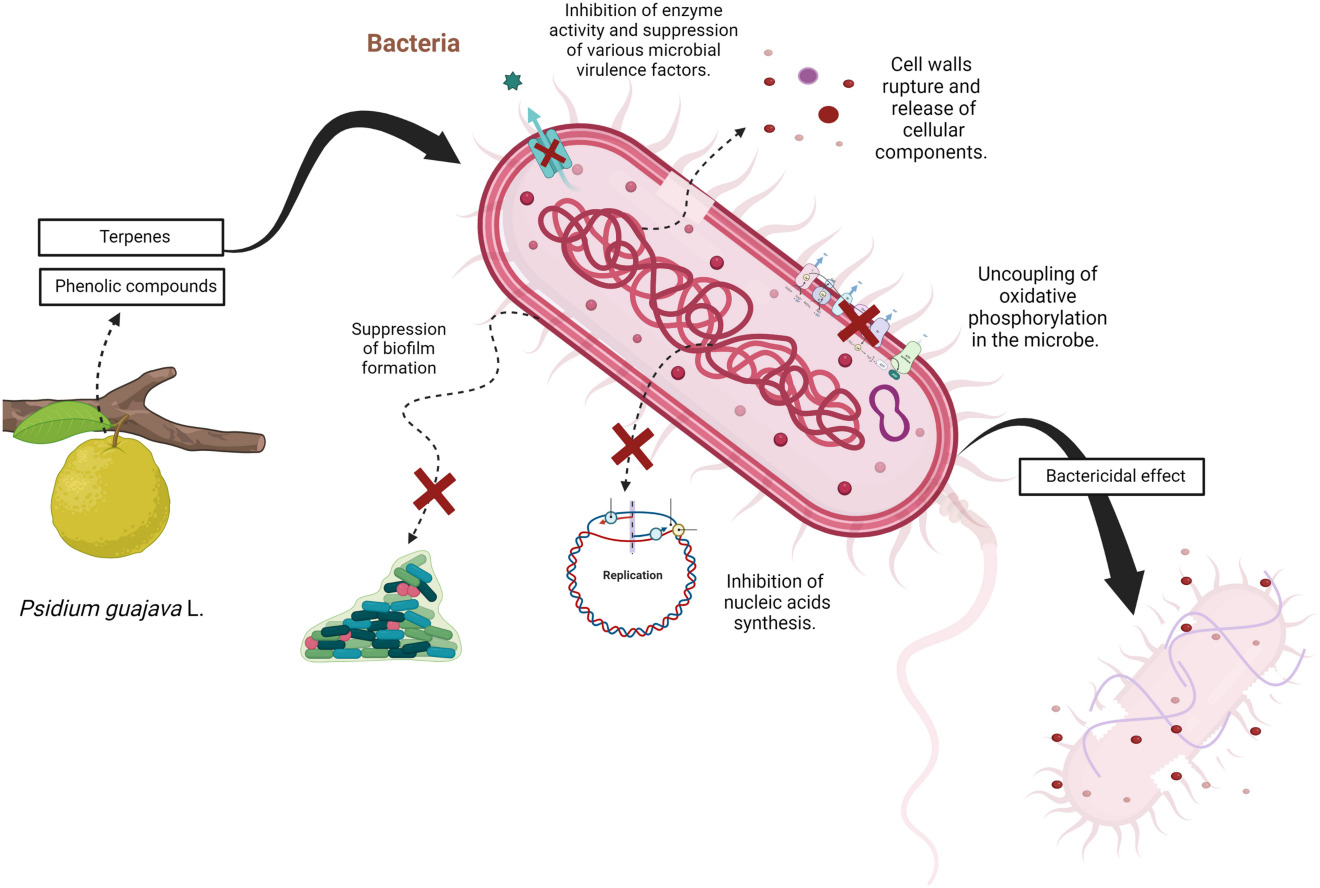
The possible antibacterial mechanisms of action of *Psidium guajava* L., adapted and modified from Bankour et al. (84).

On the other hand, the proposed mode of action of β-caryophyllene, a bicyclic sesquiterpene considered by multiple authors as the main component of guava leaf extract (45, 49, 50), is through altering the bacterial membrane permeability and causing non-selective pore formation. This induces the intracellular content leakage leading to damage and loss of the membrane integrity and may eventually lead to cell death (52). Figure 4 summarizes the possible mechanisms of action for the bactericidal activity.

Regarding the antiviral activity exerted by phenolic compounds, the mechanisms proposed are the suppression of the infection process and/or the repression of viral replication. In this regard, reported antiviral modes of action include viral DNA/protein damage and/or inhibition of viral enzymes (85).

## 8. Antidiabetic activity

Diabetes mellitus is a chronic disease that causes an increase in blood glucose levels, type 1 is caused by the loss of beta cells from the pancreatic islets, so the pancreas is no longer capable of producing insulin, while type 2, is caused by insulin resistance, so the body cannot effectively use the insulin it produces (86).

In 2021, 13% of deaths in Mexico were due to diabetes, and of the deceased, 74.9% were not insulin dependent (87). In addition, the country ranks second in Latin America with the highest prevalence of diabetes with 11.5 million cases (88).

Although, in Mexico there are different treatments available such as insulin and oral hypoglycemics, a large part of the population continues to use and even prefers the use of medicinal plants (86, 89). In addition, it should be noted that many current treatments have side effects such as gastrointestinal problems, heart failure, weight gain, edema, impaired kidney function, pancreatitis, and genital infections (90).

Multiple hydrolytic enzymes are involved in carbohydrate digestion, including α-amylase, which hydrolyzes starch, glycogen, and many oligosaccharides into maltose, maltotriose, and oligoglycans, which are further hydrolyzed by α-glucosidase to glucose, more suitable for absorption, increasing blood glucose levels (91, 92). Thus, inhibition of these enzymes can decrease postprandial glucose uptake and is therefore a key therapeutic target in the management of type 2 diabetes mellitus (91). Table 5 presents different studies that have analyzed the inhibition of these enzymes by extracts of *P. guajava* L. and their results.

**Table 5:** Inhibition of α-amylase and α-glucosidase by *Psidium guajava* L. extracts.

**Extract**	**Location**	**Enzyme**	**Results**	**References**
Ethanolic leaves extract	Madagascar	α-glucosidase, from *Saccharomyces cerevisiae*	*IC*_50_= 1.0 ± 0.3 μg/ml	(93)
Ethanolic bark extract	Madagascar	α-glucosidase, from *Saccharomyces cerevisiae*	*IC*_50_=0.5 ± 0.01 μg/mL	(93)
		α-amylase type VI-B, from porcine pancreas	*IC*_50_=10.6 ± 0.4 μg/mL	
Methanolic leaves extract (crude extract)	Japan	α-amylase, from *Bacillus sp*.	*IC*_50_= 38.0 ± 1.6 μg/mL	(94)
		α-glucosidase from *Saccharomyces sp*.	*IC*_50_= 46.0 ± 3.5 μg/mL	
Butanol-soluble fraction from methanolic leaf extract	Japan	α-amylase, from *Bacillus sp*.	*IC*_50_= 21.9 ± 0.7 μg/mL	(94)
		α-glucosidase from *Saccharomyces sp*.	*IC*_50_= 54.2 ± 3.5 μg/mL	
Fruit juice	Mexico	α-glucosidase	76.62% of inhibition	(91)
Leaves extract	Taiwan	α-amylase	*IC*_50_= 50.5 g/mL	(95)
		α-glucosidase	*IC*_50_=34.6 g/mL	

As we can see in Table 6. multiple studies have analyzed the effect of *Psidium guajava* L. on the inhibition of α-amylase and α-glucosidase, obtaining good *in-vitro* results; this may be due to the presence of quercetin in the extracts, a competitive inhibitor of α-glucosidase, since the hydroxyl present in the 3, 3′ and 4′ carbons in its structure can interact with the Asp214 and Glu276 present in the enzymatic active site (91). In addition, tannins have also been shown to be good inhibitors of α-glucosidase with Ki values similar to those of synthetic inhibitors such as acarbose and voglibose, which are currently used as treatment to control type 2 diabetes (101). The variations in the *IC*_50_values can be due to multiple factors, among them, the location and season of the year in which the biological material was collected, the extraction method, possible differences in the methodologies and in the enzymes used, for example, Beidokhti et al. (93) used a porcine α-amylase, while Samejima et al. (94) a bacterial one.

**Table 6:** Toxicological evaluation of extracts of *Psidium guajava* L.

**Extract**	**Assay**	**Animal**	**Dose**	**Results**	**References**
Bark methanolic extract	*In-vivo*, Acute and subacute toxicity	Nulliparous and non-pregnant female Wistar rats (12 weeks) for the acute toxicity Males and females (nulliparous and non-pregnant) Wistar rats (170–245 g) for the subacute toxicity test	Oral 5,000 mg/kg	Acute toxicity: no abnormality or mortality was observed	(96)
			Oral 250, 500, and 1,000 mg/kg	Subacute toxicity: variations in body weight, relative weight of organs, and biochemical parameters were observed	
Fruit ethanolic extract	*In-vivo*, Acute toxicity	Nulliparous and non-pregnant female Swiss Webster albino mice (20–30 g, 8–12 weeks)	Oral 5,000 and 2,000 mg/kg	Any differences in body weight, number of hepatocyte in liver, and podocyte in kidney compared with control	(97)
Leaves aqueous extract	*In-vivo*, Acute toxicity	Female Wistar albino rats (150–180 g, 10–12 weeks)	Oral 50, 500, and 5,000 mg/kg	Administration did not cause death of any of the animals. There was no toxicity signs, or any pathological observation	(98)
Leaves and bark aqueous extracts	*In-vivo*, Brine Shrimp Lethality Assay (BSLA)	Brine shrimp *(Artemia salina)*	1,000, 500, and 100 μg/mL	Leaves extract LC_50_ (μg/ml) = 949.13	(99)
				Bark extract LC_50_ (μg/ml) = 480.14	
Leaves extracts	*In-vivo*, Brine Shrimp Lethality Assay (BSLA)	Brine shrimp *(Artemia salina)*	–	Methanolic extract: LC_50_ (μg/ml) = 63.81 ± 2.65	(100)
				Chloroform extract: LC_50_ (μg/ml) = 41.05 ± 3.19	
				Hexane extract: LC_50_ (μg/ml) = 32.18 ± 0.24	

Xu et al. (102) administered 800 mg/kg of guava leaf extract daily to diabetic rats and observed a significant reduction in total cholesterol, Low-density lipoprotein cholesterol (LDL-C), and glycosylated serum protein (precise intermediate marker of glycemia) compared with the control group (diabetic rats that received only distilled water). Exposing that guava leaves have antihyperglycemic and antihyperlipidemic effects.

The same year, Luo et al. (103) worked with diabetic mice induced by streptozotocin and high-fat diets and reported an improvement in body weight, decrease in fasting blood glucose, total triglycerides and total cholesterol levels with the daily intragastric administration of polysaccharides isolated from guava leaves (100 and 200 mg/kg) for 4 weeks. They even observed protective effects on the kidney, since the creatine content, whose excretion is an indicator of the organ's metabolism and whose accumulation indicates impaired functioning, decreased after the administration of the polysaccharides. Similarly, Glycated serum protein levels decreased in a dose-dependent manner.

On the other hand, Eidenberger et al. (104) analyzed the absorption of guava leaf extract in the CaCo-2 cell line, widely used to investigate nutrient absorption, and concluded that the extract can exert its observed effect *in-vitro* after being administered orally. Although there are results that indicate a potential antidiabetic effect, there is still much to be investigated, especially the activity and safety in humans, the possible mechanisms of action and the long-term effects.

## 9. Toxicity

Numerous health benefits of plants and their extracts have been reported, for example, as potential antioxidant, anticancer, antimicrobial, and anti-inflammatory agents. However, even though they may exhibit excellent *in-vitro* activity, we cannot take advantage of these benefits until they are proven to have no harmful side effects. The use of a plant for a certain purpose must not only be effective, but also safe for the consumer (105).

*In-vivo* and *in-vitro* toxicology studies are conducted to determine a drug's short and long term functional and morphologic adverse effects. There is a large variety of toxicological studies, among them, the acute toxicity studies are carried out to determine the short-term adverse effects of a single dose of a drug (or multiple doses during a period of 24 h) and the subacute toxicity studies, performed to evaluate the possible adverse effects of a new drug after a treatment period of 2–4 weeks duration (106). Table 6 shows some of the toxicological studies carried out with guava extracts.

Manekeng et al. (96) evaluated the toxicity of orally administered *Psidium guajava* L. bark methanolic extract in Wistar rats, the authors observed no abnormalities or mortality in rats at a dose of 5,000 mg/kg, however, they mention that the repeated administration of high doses (1,000 mg/kg or more) could exhibit mild organ toxicity. Similarly, Atik et al. (97) in their acute toxicity test observed no side effects in Swiss Webster mice after oral treatment with 2,000 and 5,000 mg/kg of guava ethanolic extract.

Babatola et al. (98) evaluated the acute toxicity of aqueous extracts of guava leaves of three different varieties (white, red, and pink guava), none of the extracts caused death or generated any symptoms of pathology at concentrations of 50, 500, and 5,000 mg/kg. These results are interesting because we must remember that the composition of the extracts can vary due to many factors, among them the variety and therefore, the toxicity can also be affected; this study was not the case, but it is important to consider this fact to avoid generalizing the results.

Bautista et al. (99) conducted the Brine Shrimp Lethality Assay (BSLA), a simple and inexpensive bioassay used for evaluating the efficacy of phytochemical present in the plant extracts. This assay is based on the ability to kill a simple zoological organism on 24 h (107). The authors evaluated the toxicity of aqueous extracts of guava bark and leaves and concluded that bark leaf extract is more toxic compared to leaf extract.

The toxicological evaluation is very important, especially considering that in many parts of the world, including Mexico, it is thought that herbal products, thanks to their natural origin, are not toxic and are used indiscriminately, nonetheless, everything depends on the dose and the consumer. Unfortunately, in recent years an increase in cases of adverse effects of herbal products has been reported (98), which reflects the need for better and more complete toxicological evaluations of plant extracts.

## 10. Bioavailability

Bioavailability is the ability of certain compound to reach the circulatory system and be distributed throughout the body. While bioaccessibility, another important concept, refers to the amount of compound that is available for absorption in the gastrointestinal tract (108).

The digestion of phenolic compounds is a complex and not fully understood process, however, today we know that the release of phenolic compounds begins with mastication (chewing) and the low pH of the stomach, which generates the disintegration of the food matrix. Subsequently, depolymerization begins, also in the stomach, where the phenolic compounds are degraded into small structures so that they can be absorbed. Uptake of small phenolic compounds, particularly aglycones, occurs by passive diffusion (lactase phloridizin hydrolase activity) or active transport by sodium-dependent glucose transporter (cytosolic β-glucosidase). Unabsorbed phenolic compounds now move to the colon, where the gut microbiota can facilitate absorption of phenolic compounds, nevertheless, the variability within individual gut microbiota is a determinant factor to increase the bioaccessibility of phenolic compounds (108).

The bioavailability of phenolic compounds depends on several factors, including release from the matrix during gastrointestinal digestion, bioaccessibility, cellular uptake, metabolism, and further transport in the circulatory system. Unfortunately, most phenolic compounds are poorly absorbed in the small intestine, as they are either heavily metabolized or rapidly eliminated. In addition, polyphenols can be associated with the dietary fiber matrix, through covalent bonds or hydrophobic interactions, which means that they are not bioavailable in the human intestine (109).

Similarly, terpenes have low water solubility and low bioavailability. Furthermore, it has been noticed that terpenes have a highly lipophilic behavior that influences their solubility in the aqueous phase of the intestinal lumen and, therefore, their bioavailability according to their incorporation into a lipid phase, either during digestion or during food processing (110).

To enable phytochemicals compounds, like polyphenols and terpenes, applications as antimicrobial agents, it is necessary to improve its bioavailability. One of the most promising strategies, which can also increase their stability, is the use of delivery systems, such as liposomes, nanoemulsions, and polymeric/biopolymeric nanoparticles (111). For example, Vasconcelos et al. (112) generated a self-emulsifying drug delivery system containing purified lycopene from red guava.

## 11. Clinical trials

Birdi et al. (113) conducted a clinical trial with 109 patients diagnosed with diarrhea. The authors reported that a decoction of guava leaves was a safe and simple treatment for common diarrhea. However, some trial limitations must be considered, first, the study was conducted with adults, so the safety and effectiveness in children, who are more susceptible to this pathology, remains to be proven. Moreover, since bacteriological examination of the stools was not undertaken at screening to identify causative organism, the laboratory results could not be verified clinically from the patients in this trial.

In addition, Nayak et al. (114) conducted a double blind randomized, placebo controlled clinical trial with subjects with moderate to severe chronic gingivitis. The authors evaluated the potential activity of an hydroethanolic extract of guava leaves incorporated in a mouthrinse in 56 patients aged between 18 and 40 years who were evaluated for 3 months. Although the guava mouthrinse group showed beneficial results, like reductions in plaque scores and improvement in gingival health, the results were not statistically significant. In addition, further randomized clinical trials should be carried out for an extended period to substantiate its long-term effects.

Similarly, Amaliya et al. (115) evaluated the effect of guava and vitamin C supplementation on gingivitis. The randomized clinical trial had the participation of 48 persons; however, it was also carried out in a short period of time (4-week) which did not allow to make firm conclusions.

Pongsakornpaisan et al. (116) conducted a study on the efficacy of their anti-sebum toner based on guava leaf extract with 10 volunteers. The authors observed that guava toner suppressed the sebum level in the nose and forehead area after 28 days of treatment and concluded that guava may be a good agent for cosmetic products. Although, the number of participants was small and long-term effects need to be assessed.

König et al. (117) reported that guava extract obtained by supercritical *CO*_2_ extraction reduced postprandial blood glucose levels in a randomized, double-blind, parallelized clinical study conducted with 31 young and healthy participants (20 female and 11 male, ages 19–29) from the Paracelsus Medical University Salzburg. After an overnight fast, each participant underwent an oral glucose tolerance test, in which members of the control group each received a glucose solution (75 g glucose) and members of the intervention group each received a glucose solution that also contained 2.5 ml of extract. However, it should be considered that the number of participants was small and only healthy young people were considered, so the effect on adults, children, and people with chronic diseases, including diabetes, is unknown. In addition, a low concentration of the extract was used, so possible side effects at higher concentrations and after long periods of administration were not reported. An important fact also mentioned by the authors is that the effect of the extract decreased after storage, therefore, it is necessary to consider the possible instability of the extracts over time for future applications.

Another similar clinical trial was carried out by Kumari (118), which had 45 young and healthy participants (31 male and 14 females, 18–25 years). The trial was conducted for 4 months, with 6 weeks of guava supplementation. Fifteen subjects were supplemented with 400 g of ripe guava with peel, another 15 with 400 g guava without peel and 15 subjects were the control group, which did not receive any supplement. After supplementation with ripe guava without peel a drop in blood sugar was observed. The authors attribute this effect to the high fiber content in the guava pulp, since it delays the intestinal absorption of glucose; the presence of flavonoid glycosides that improve insulin sensitivity, as well as the inhibition of the α-glucosidase enzyme. In addition, there was also a decrease in total cholesterol and triglycerides and an increase in high-density lipoproteins (HDLc). However, supplementation with ripe guava fruits with peel gave different results, since it increased blood glucose levels, cholesterol, triglyceride, and low-density lipoprotein (LDLc) levels. According to the authors, this effect may be since the peels have a low concentration of magnesium, necessary for the activity of multiple enzymes and which favors insulin-dependent glucose absorption; however, this explanation is ambiguous, and more studies are necessary to clarify this phenomenon.

More detailed clinical trials need to be conducted to establish the efficacy of the guava extracts.

## 12. Perspectives and conclusions

The potential of *Psidium guajava* L. and its different extracts is evident, however, there is still much to investigate so the different benefits can have an industrial application and therefore can reach society.

First, the repertoire of strains used in the analysis of antibiofilm effects must be expanded, moreover, most of the published studies about antimicrobial effects are *in*-*vitro* evaluations (antibiograms, microdilution method, etc.), therefore, *in-vivo* studies should be encouraged in the coming years.

On the other hand, the literature focuses mostly on the bioactivity of guava leaves, however, if the aim is to use agro-industrial residues, it will probably be difficult to separate the leaves from the rest of the waste (heterogeneous mixture of leaves, bark, seeds, and pulp), so it would be interesting to analyze the effect of an extract obtained from this combination. Another inconvenience that should be considered when using waste and byproducts from the agroindustry is the possible presence of food additives and pesticides.

Besides, it is important to improve the long-term stability of the extract, since in most studies the conservation of the extracts is carried out by freezing, refrigeration, or drying, which would not be very viable in industrial applications. In addition, it is known that phenolic compounds have low bioavailability, so it is necessary to find a way to protect them and make them more available, for example, through encapsulation.

More detailed clinical trials need to be conducted to establish the efficacy of guava extracts, and it would be interesting to analyze their effect in the treatment of other diseases than diarrhea or oral problems, for example, flu and acne.

Finally, the possible variation in phytochemicals present in the extracts due to changes in climate, season, and other factors such as the presence of insects can cause a lack of homogeneity in the final product, which should also be considered if commercialization is sought.

The recovery and valorization of guava waste can bring economic benefits to the agroindustry and can represent an alternative to the use of traditional antimicrobials, helping the problem of microbial resistance in Mexico, which has easy access to this residue. In addition, it can reduce environmental problems caused by leaching and emission of greenhouse gases generated during the treatment of agro-industrial waste.

## Author contributions

DGM was the main writer of the review. ALGB is the main head of the laboratory. NACV and FJAG review the manuscript. IGOG also participate in the redaction of the review. All authors contributed to the article and approved the submitted version.
